# The Scientific American

**Published:** 1880-01

**Authors:** 


*The Scientific American.* Published by Munn & Co., 37 Park Row, New York City, for $3.20, postage included, per annum, has now reached a weekly edition of fifty thousand copies. Its contents are varied and instructive, comprising much that is useful and interesting, together with finely executed engravings of machinery, useful inventions, and natural history.

Every number contains interesting articles on scientific subjects, from which much useful information can be derived, and no expense is spared to render it attractive as well as useful to all professions and trades.

